# pyALRA: python implementation of low-rank zero-preserving approximation of single cell RNA-seq

**DOI:** 10.1093/bioadv/vbaf279

**Published:** 2025-11-09

**Authors:** Alexandre Lanau, Joshua J Waterfall

**Affiliations:** INSERM U1330, Institut Curie Research Center, PSL Université, 26 rue d’Ulm, Paris, 75005, France; Translational Research Department, Institut Curie Research Center, PSL Université, 26 rue d’Ulm, Paris, 75005, France; INSERM U1330, Institut Curie Research Center, PSL Université, 26 rue d’Ulm, Paris, 75005, France; Translational Research Department, Institut Curie Research Center, PSL Université, 26 rue d’Ulm, Paris, 75005, France

## Abstract

**Motivation:**

Some recently published methods for single-cell RNA-seq preprocessing and correction are not necessarily available in both Python and R, which limits the accessibility of these tools to the wider community.

**Results:**

We present pyALRA, an efficient python implementation of the (r-)ALRA R package conceived to impute drop out values using a low-rank zero-preserving approximation for single cell RNA-seq. This re-implementation achieves similar prediction performance using corresponding python methods and allows both speed and RAM consumption improvements.

**Availability and implementation:**

pyALRA is released as an open-source software under the MIT license. The source code is available on GitHub at https://github.com/alexandrelanau/pyALRA and on Zenodo at https://doi.org/10.5281/zenodo.15730914.

## 1 Introduction

Single-cell RNA sequencing (scRNA-seq) has revolutionized transcriptomic research by enabling the measurement of gene expression at the individual cell level, providing invaluable insights into cellular heterogeneity and gene regulation. However, a prominent challenge in analyzing scRNA-seq data is the high prevalence of false zeros or “dropout” events, where genes that are actually expressed may appear as unexpressed due to undersampling or other technical limitations. These dropout events can significantly impede downstream analyses, such as clustering and differential expression, necessitating robust imputation methods that can accurately recover the underlying biological signal while preserving the true biological zeros (genes not expressed at the time point of library generation).

Various approaches have been developed to address this issue, including deep learning-based methods like DCA (Eraslan *et al.* 2019), graph-based techniques such as MAGIC (Van Dijk *et al.* 2018), and statistical models like SAVER (Huang *et al.* 2018). While these methods have shown improvements in imputing technical zeros, many fail to preserve true biological zeros, potentially leading to overestimation of gene expression across cell populations (Linderman *et al.* 2022). ALRA (Adaptively thresholded Low-Rank Approximation) (Linderman *et al.* 2022) was introduced to overcome these limitations by selectively leveraging the non-negativity and low-rank structure of the true expression matrix, which is assumed to still contain many biological zeros. It first computes a low-rank approximation using singular value decomposition (SVD), selecting the rank k based on the distribution of the eigenvalues. Specifically, k is chosen as the first rank where the difference between successive eigenvalues sk satisfies sk > μ + 6σ, with μ and σ denoting the mean and standard deviation of these differences, respectively. Following the low-rank reconstruction, biological zeros are restored by thresholding each gene’s values using the symmetry of the error distribution inferred from the negative entries. The resulting method, r-ALRA, has been shown to outperform existing imputation techniques while offering greater computational efficiency (Linderman *et al.* 2022).

However, ALRA is currently available only in R despite the growing use of python for single cell analyses, such as with the recent development of the scverse ecosystem (Virshup *et al.* 2023). Existing methods for interfacing R and Python, such as rpy2 (https://rpy2.github.io/), are often time-consuming and require significant effort, while offering limited control over the interaction between the two languages. Effectively, while interfacing Python and R can be useful when no Python-native implementation exists, it introduces an additional layer of complexity on top of already memory-intensive processes. As mentioned, when **rpy2** interfaces with R, it creates a copy of the object. This not only increases RAM consumption but also slows down execution and can lead to incompatibilities or mismatches between the two languages and data structures.

Here, we present a direct Python implementation of the ALRA methodology for scRNA-seq data imputation, aiming to make this tool easily and widely accessible to the Python-based bioinformatics community. Our implementation facilitates integration with modern Python data science frameworks, enhances computational scalability, and provides results consistent with the original method. Through benchmarking on multiple datasets, we demonstrate that our implementation achieves comparable performance to existing methods while preserving biological zeros, thus improving the reliability of downstream scRNA-seq analyses. (pyALRA and r-ALRA are developed by independent groups).

## 2 Implementation

We applied the Adaptively thresholded Low-Rank Approximation (ALRA) method to several single-cell RNA sequencing (scRNA-seq) datasets to evaluate its performance in imputing expression values while preserving biological zeros. In this implementation, we leveraged only well-maintained packages: scipy = 1.14.0, scikit-learn = 1.5.2, numpy = 1.26.4.

### 2.1 Available features and code structure

pyALRA normalizes the input expression matrix by library size and applies a log transformation to stabilize variance, tailoring normalization to the data type. It uses the randomized_svd function from the sklearn.utils.extmath module to perform randomized singular value decomposition (SVD), adaptively determining the rank of the low-rank approximation based on the distribution of singular value differences. As described in the original r-ALRA publication, the rank k that represents biological signals from the SVD is identified as the lowest singular value (SV) which is larger than the subsequent SV by an amount significantly greater than the differences between SVs in the tail of the distribution (by default lowest 20 SVs). The process reconstructs the matrix and applies a quantile-based thresholding method to impute missing values while preserving biological zeros, enhancing the reliability of downstream analyses.

### 2.2 Benchmark of pyALRA versus r-ALRA package

**Figure 1.**
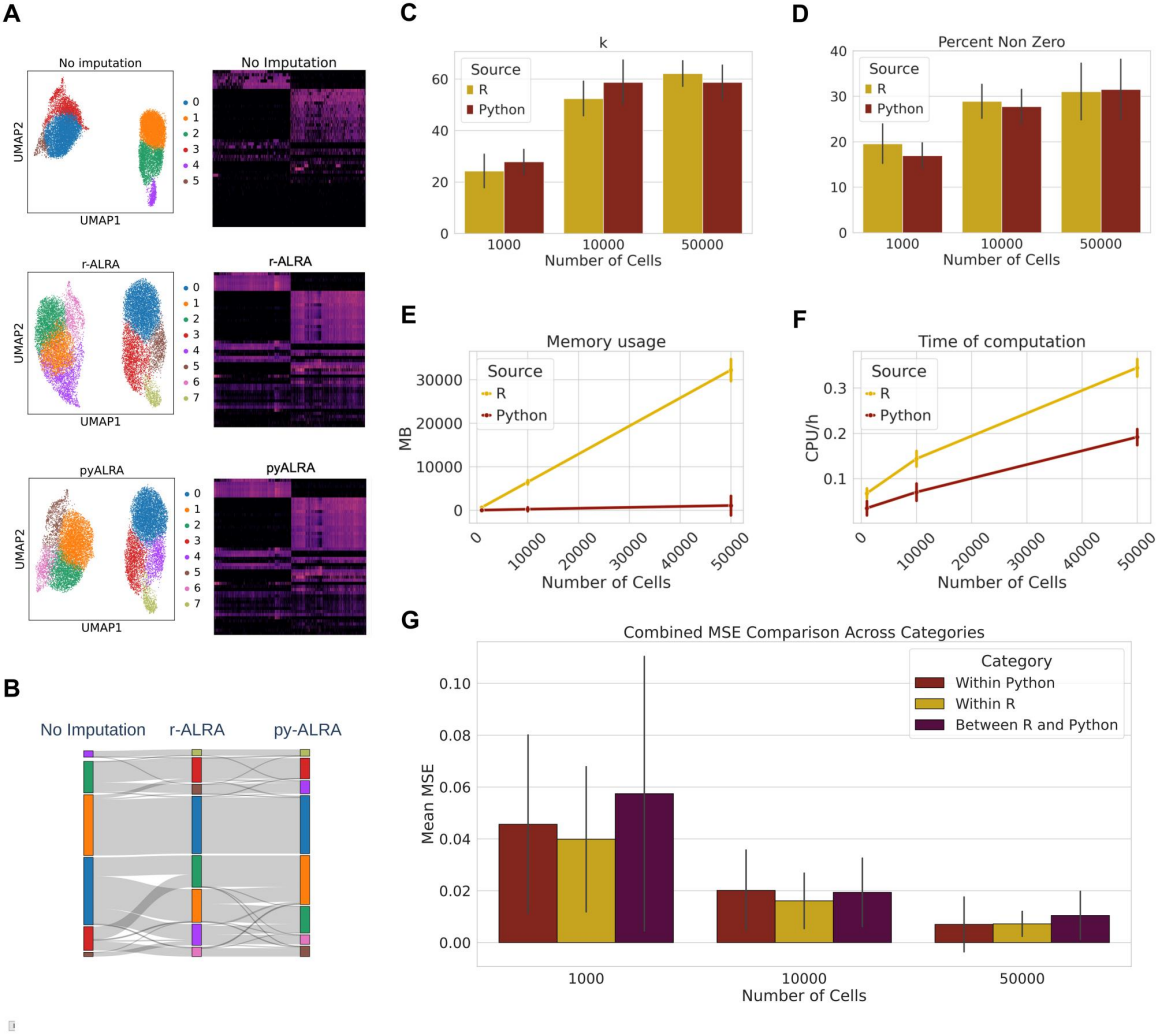
pyALRA presents similar predictions with better performance. (A) UMAP of single cell RNA sequencing from r-ALRA PBMC tutorials (Linderman *et al.* 2022) and associated heatmaps of 50 highly variable genes, only normalized (top panel), r-ALRA processed (middle panel) or pyALRA processed (bottom panel). (B) Alluvial plot of cluster composition across the different imputation conditions from (A). (C) Comparison of k predicted using randomized SVD algorithm between R and Python implementation (error bars = standard deviation). (D) Comparison of non-zeros genes predicted between R and Python implementation (error bars = standard deviation). Comparison of Python and R implementation of ALRA for RAM usage (Mb) (E) and CPU/h (F) (error bars = standard deviation). (G) Mean-squared error comparison between pyALRA and ALRA implementation (error bars = standard deviation). For (C–G), all results are from five runs in subsamples (1000, 10 000, 50 000 cells) from three independent datasets.

To evaluate the relevance of our implementation, we measured prediction performance and computational resource consumption. All predictions are based on the same input matrices, which were previously normalized (e.g. by library size and log-transformed). As qualitative evidence, we used the PBMC dataset from r-ALRA tutorials to compute UMAP with Leiden clustering and heatmaps of the top 50 highly variable genes based on normalized data (i) without imputation, (ii) r-ALRA imputed, or (iii) pyALRA imputed values (Fig. 1A). r-ALRA and pyALRA present qualitatively similarly shaped UMAPs and clustering (with regards to seed variation in UMAP). Using alluvial plot, we can identify high sharing of cluster composition between r-ALRA and pyALRA imputed clusters (Fig. 1B). Finally, the improvement in dropout count recovery by both implementations, as well as their similarity in predictions, is clearly visually apparent by the heatmaps, even compared to another python-native algorithm (MAGIC) (Fig. 1A, Fig. 1, available as supplementary data at *Bioinformatics Advances* online). To assess the biological relevance of pyALRA predictions, we reprocessed a thymic epithelial cell (TEC) dataset(Park *et al.* 2020) to evaluate expression correction on tissue-restricted antigens (TRAs) (Fig. 2, available as supplementary data at *Bioinformatics Advances* online). We computed UMAP and Leiden clustering, as in Fig. 1A, to compare the impact of correction and found that pyALRA produced embeddings similar to those reported in the original publication (Fig. 2A and B, available as supplementary data at *Bioinformatics Advances* online). Finally, the expression of the key TEC transcription factor **AIRE**, as well as several TRAs (**INS, GFAP, MYO7A**), was better recovered using pyALRA compared to the other methods (Fig. 2C, available as supplementary data at *Bioinformatics Advances* online).

To perform quantitative comparisons, when comparing the predicted low dimensional rank (k values) from the randomized SVD method, r-ALRA and pyALRA present similar results (Fig.1C, Figs 3–5, available as supplementary data at *Bioinformatics Advances* online). Moreover, when comparing the prediction of non-zero genes by r-ALRA and pyALRA, both methods show similar percentages of predicted non-zero genes (i.e. from zero in the original data toward a non-zero value after correction) across datasets (Fig. 1D, Figs 3–5, available as supplementary data at *Bioinformatics Advances* online).

Additionally, we compared the computational requirements between the R and Python implementations of the algorithm. pyALRA consistently demonstrates better performance, both in terms of memory usage and time requirement (Fig. 1E and F, Figs 3–5, available as supplementary data at *Bioinformatics Advances* online).

To explore the robustness of our implementation as a function of dataset size, we randomly subsampled three subsets of increasing size (1000, 10 000, and 50 000 cells) from three separate datasets. For each subset, five independent runs were performed. Analyzing the mean-squared error of predicted counts between each run for each dataset, within python predictions, within R predictions and between both implementations, we observed highly consistent results across implementations (Fig. 1G, Fig. 6, available as supplementary data at *Bioinformatics Advances* online). In summary, our python implementation of the ALRA algorithm generates equivalent results to the original R implementation with dramatically improved time and memory consumption.

## 3 Conclusion

In conclusion, pyALRA is an efficient and reliable method to impute technical zeros in single-cell RNA-seq data. It produces equivalent output to the original R implementation with significant improvements in time and memory usage. The new implementation facilitates integration into python workflows, and the improved computational performance allows analysis of larger datasets. However, dropout inference methods, such as ALRA, aim to recover missing gene expression values caused by technical noise, but their effectiveness can be highly context-dependent, varying with dataset characteristics like sequencing depth, cell type heterogeneity, and the underlying biological sparsity.

## Supplementary Material

vbaf279_Supplementary_Data

## Data Availability

The data underlying this article were downloaded from EBI Single Cell Expression atlas. (www.ebi.ac.uk/gca/sc/experiments), using scanpy.datasets.ebi_expression_atlas function from scanpy. Following datasets were used: E-MTAB-8142, E-MTAB-7407, E-GEOD-139324.
