# Supplementary material for: pyALRA: python implementation of low-rank zero-preserving approximation of single cell RNA-seq
Source: Bioinform Adv. 2025 Nov 9;5(1):vbaf279. doi: 10.1093/bioadv/vbaf279 (PMC12664701; doi:10.1093/bioadv/vbaf279)
Supplement: vbaf279_Supplementary_Data [file vbaf279_supplementary_data.zip › pyALRA_fig_reviewingR2_figS5.pdf]

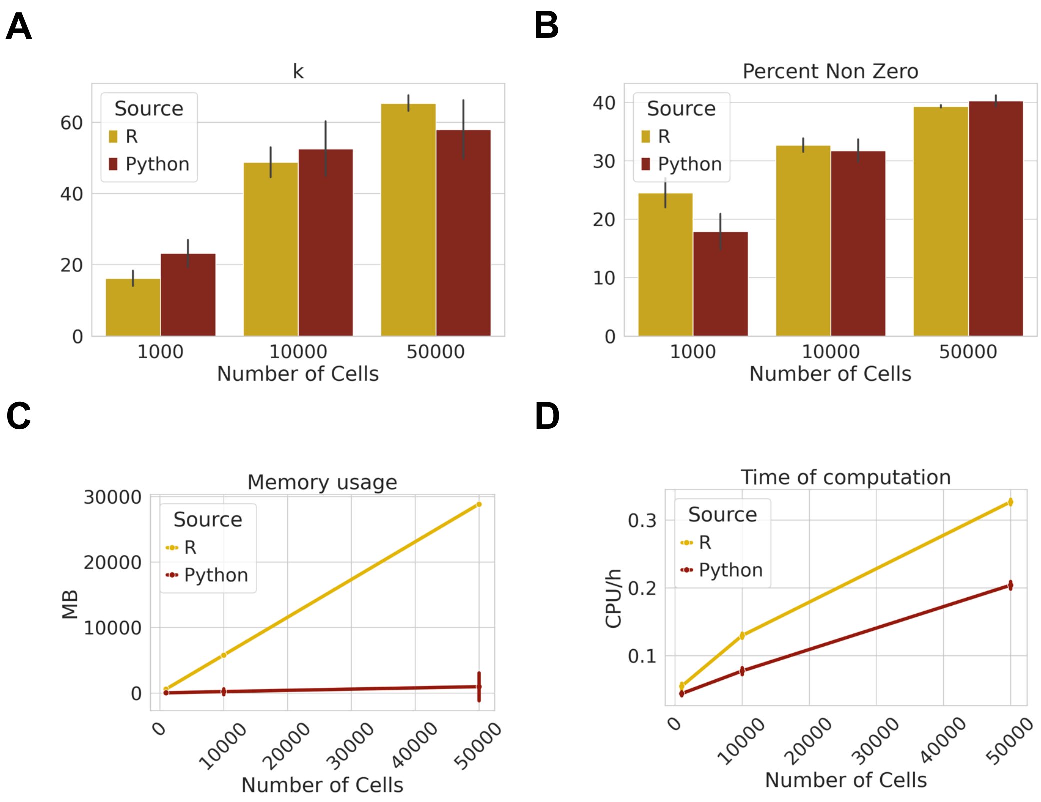

Figure S5: Performance of prediction for E-GEOD-139324

(**A)** Comparison of k predicted using randomized SVD algorithm between R and Python implementation (n=15, error bars = standard deviation). **(B)** Comparison of non-zeros genes predicted between R and Python implementation (n=15, error bars = standard deviation). Comparison of Python and R implementation of ALRA for RAM usage (Mb) (**C)** and CPU/h **(D)** (n=15, error bars = standard deviation).
