# Supplementary material for: pyALRA: python implementation of low-rank zero-preserving approximation of single cell RNA-seq
Source: Bioinform Adv. 2025 Nov 9;5(1):vbaf279. doi: 10.1093/bioadv/vbaf279 (PMC12664701; doi:10.1093/bioadv/vbaf279)
Supplement: vbaf279_Supplementary_Data [file vbaf279_supplementary_data.zip › pyALRA_fig_reviewingR2_figS6.pdf]

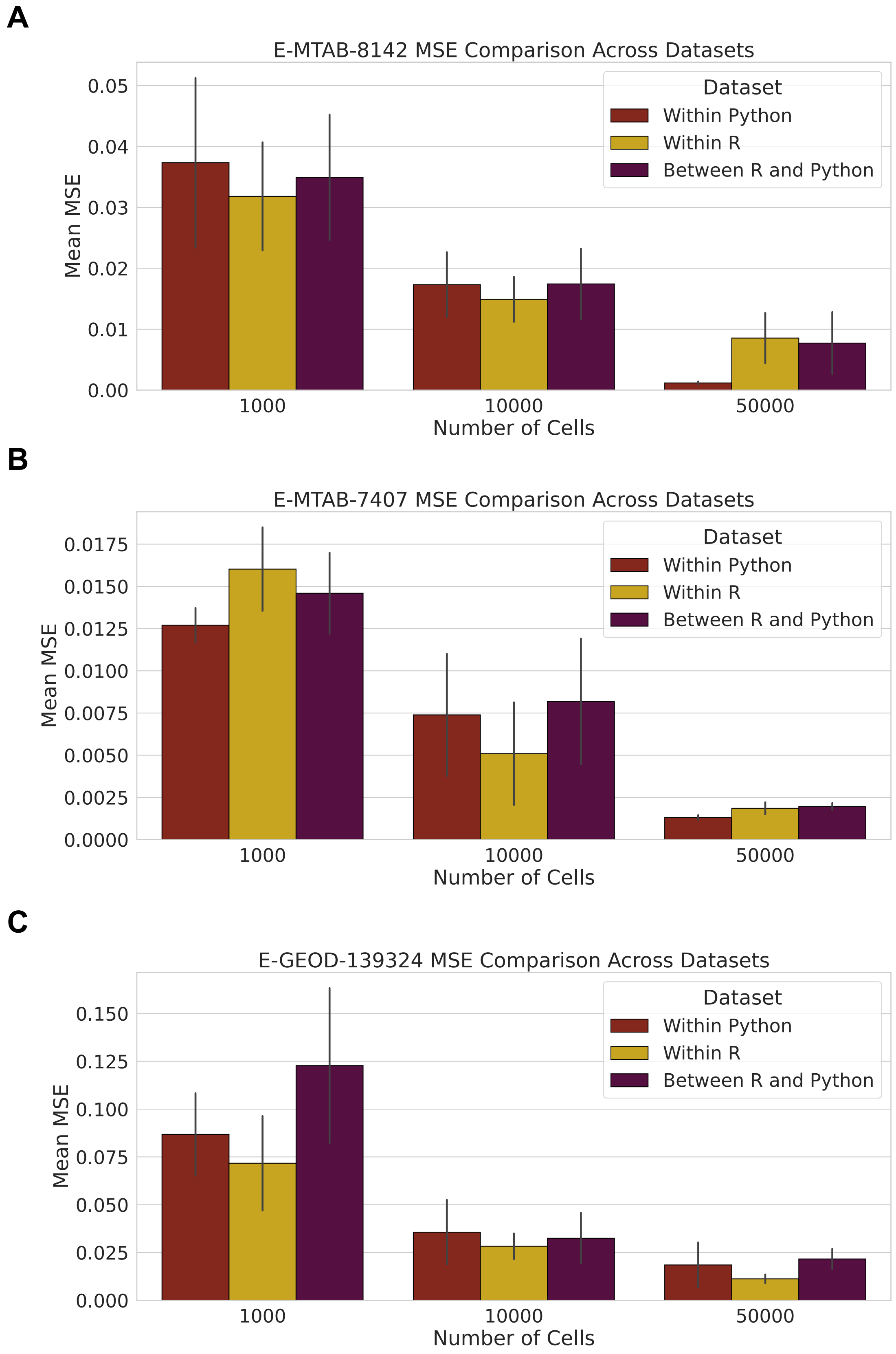

Figure S6: Pairwise comparisons between pyALRA and r-ALRA runs for E-MTAB-8142, E-MTAB-7407, E-GEOD-139324

(**A-C)** Pairwise comparison within (R and between R and Python ALRA implementation (right column), and within each implementation (python – left column, R – middle column) , using mean-squared error, for several dataset sizes - 1000 **(A)**, 10000 **(B)** and 50000 cells **(C)** - and between each run.
