# Supplementary material for: pyALRA: python implementation of low-rank zero-preserving approximation of single cell RNA-seq
Source: Bioinform Adv. 2025 Nov 9;5(1):vbaf279. doi: 10.1093/bioadv/vbaf279 (PMC12664701; doi:10.1093/bioadv/vbaf279)
Supplement: vbaf279_Supplementary_Data [file vbaf279_supplementary_data.zip › pyALRA_fig_reviewingR2_figS1.pdf]

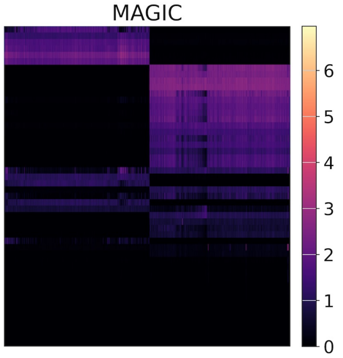

Figure S1: Heatmaps of the 50 top Highly variables genes of MAGIC imputation (python-native) from PBMC dataset
