# Supplementary material for: pyALRA: python implementation of low-rank zero-preserving approximation of single cell RNA-seq
Source: Bioinform Adv. 2025 Nov 9;5(1):vbaf279. doi: 10.1093/bioadv/vbaf279 (PMC12664701; doi:10.1093/bioadv/vbaf279)
Supplement: vbaf279_Supplementary_Data [file vbaf279_supplementary_data.zip › pyALRA_fig_reviewingR2_figS2.pdf]

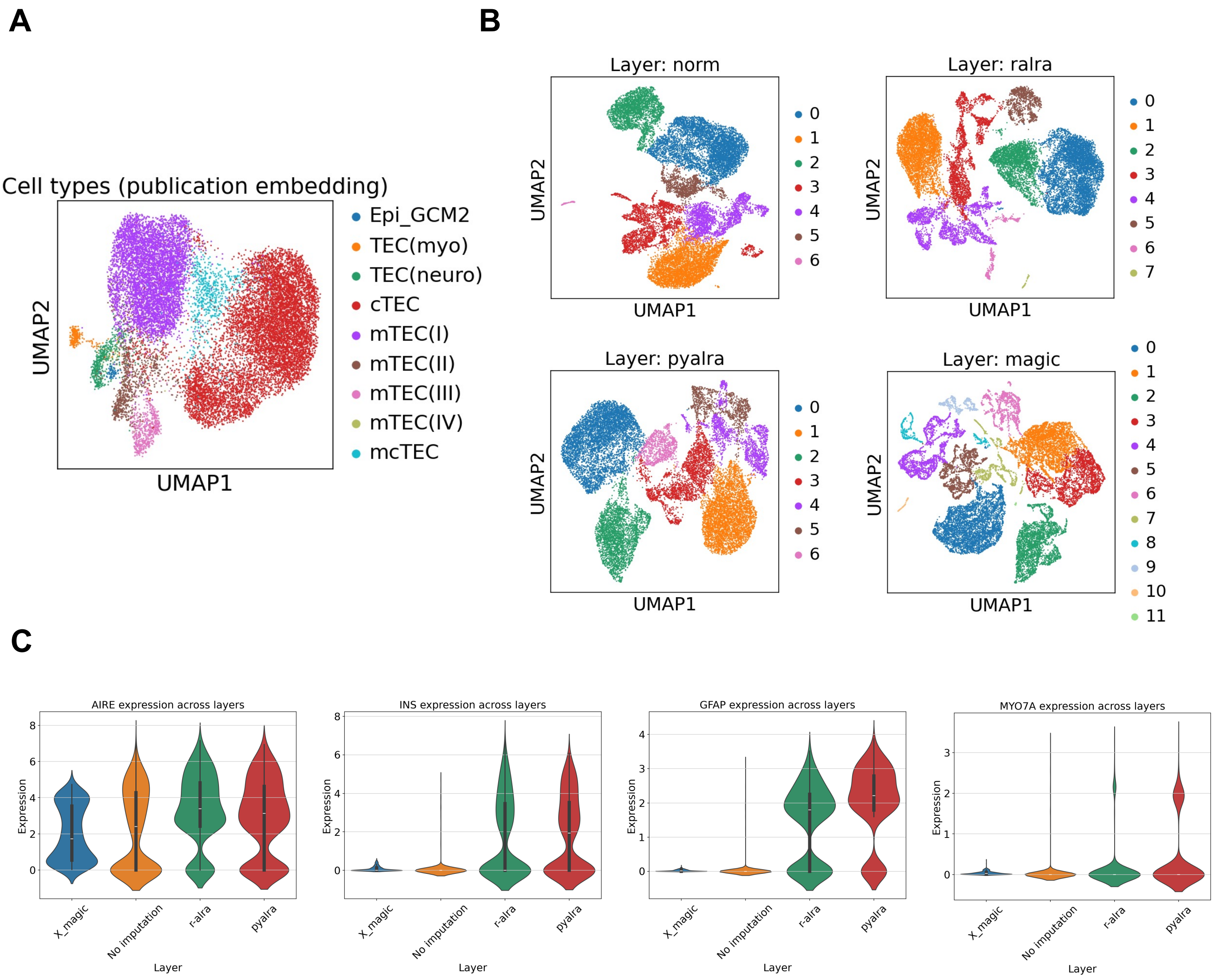

Figure S2: Imputation method comparisons on thymic epithelial cells

(**A)** UMAP of single cell RNA sequencing of TECs, with publication embedding, colored by cell type. **(B)** UMAP of single cell RNA sequencing of TECs only normalized (top left panel), r-ALRA processed (top right panel), or pyALRA processed (bottom left panel) or MAGIC processed (bottom right panel). **(C)** Comparison of expression of keys Transcription factors (AIRE) or tissue restricted antigens (INS, GFAP, MYO7A) among the mature mTEC (mTEC(II)).
